# Hand Gesture Recognition Using FSK Radar Sensors

**DOI:** 10.3390/s24020349

**Published:** 2024-01-06

**Authors:** Kimoon Yang, Minji Kim, Yunho Jung, Seongjoo Lee

**Affiliations:** 1 Department Semiconductor Systems Engineering, Sejong University, Gunja-dong, Gwangjin-gu, Seoul 05006, Republic of Korea; ykm3563@itsoc.sejong.ac.kr (K.Y.); minji@itsoc.sejong.ac.kr (M.K.); 2 Department of Convergence Engineering of Intelligent Drone, Sejong University, Gunja-dong, Gwangjin-gu, Seoul 05006, Republic of Korea; 3 Department of Smart Drone Convergence, Korea Aerospace University, Goyang 10540, Gyeonggi-do, Republic of Korea; yjung@kau.ac.kr; 4 School of Electronics and Information Engineering, Korea Aerospace University, Goyang 10540, Gyeonggi-do, Republic of Korea; 5 Department Electrical Engineering, Sejong University, Gunja-dong, Gwangjin-gu, Seoul 05006, Republic of Korea

**Keywords:** human–computer interaction, hand gesture recognition, micro-Doppler signature, FSK radar, Doppler radar, convolutional neural network, data preprocessing

## Abstract

Hand gesture recognition, which is one of the fields of human–computer interaction (HCI) research, extracts the user’s pattern using sensors. Radio detection and ranging (RADAR) sensors are robust under severe environments and convenient to use for hand gestures. The existing studies mostly adopted continuous-wave (CW) radar, which only shows a good performance at a fixed distance, which is due to its limitation of not seeing the distance. This paper proposes a hand gesture recognition system that utilizes frequency-shift keying (FSK) radar, allowing for a recognition method that can work at the various distances between a radar sensor and a user. The proposed system adopts a convolutional neural network (CNN) model for the recognition. From the experimental results, the proposed recognition system covers the range from 30 cm to 180 cm and shows an accuracy of 93.67% over the entire range.

## 1. Introduction

Human–computer interaction (HCI) is a research field that develops various interactions between humans and computers [1,2]. Typically, a keyboard and mouse have been used for human–computer interactions. However, research in regard to developing simpler interaction methods, such as voice and gesture recognition, is rapidly progressing, with the development of hardware and software in recent years [3]. Hand gesture recognition is one of the most effective human recognition methods among the various recognition techniques. Various technologies are still being widely studied on the Internet of Things (IoT) [4]. A remote control without a remote controller in a smart home environment is one of the important research areas [5,6]. This type of remote control includes various user interfaces, such as human gestures, the voice, the iris, and fingerprints. Human gestures are the simplest and most natural gestures among them. They are also the most intuitive in a smart environment, as they can interact with devices as well as be universally used in various applications. User demands about hand gesture recognition have recently been continuously increased, and the research is being actively developed with regard to improving the gesture-recognition rate [7,8]. The hand gesture system is being applied to various devices, such as drones, robots, and smart home devices, as hand gesture recognition develops [9,10].

Hand gesture recognition detects and analyzes hand movements, which can control a device using a predetermined movement pattern. Sensors are essential devices for hand gesture recognition, which mainly involve camera sensors, acceleration sensors, and radio detection and ranging (RADAR) sensors [11,12]. The recognition based on camera sensors is widely and easily conducted by photographing a hand gesture and by using vision technology. However, a vision sensor includes an infringement of personal information, and it is heavily influenced by the surrounding environment, such as low illuminance. Accelerometers have the advantage of recognizing the minute movements of hand gestures or being strong against environmental factors. However, they have the inconvenience of constantly being worn by the user [11]. On the other hand, radar sensors are hardly affected by the recognition environment, and they are relatively free from the infringement of personal information [13,14]. In addition, there is no need to wear a sensor, so radar sensors are useful for IoT services, such as smart homes.

The previous studies based on the radar sensors mainly adopted the continuous-wave (CW) radar. However, the CW radar cannot measure the distance, so there is a problem with a low recognition rate when the distance between a radar sensor and a user is changed [15]. The restricted utilization by working at a fixed distance is critical to the development of commercial products and services [16,17,18]. The algorithm proposed in this paper can address this problem by obtaining distance information via radar. There are three radar options for obtaining distance information: the frequency-shift keying (FSK) radar, frequency-modulated continuous-wave (FMCW) radar, and stepped-frequency continuous-wave (SFCW) radar. The FSK radar has the least hardware complexity and is the most cost-effective among the three radars. Moreover, it requires simpler signal processing, resulting in a relatively lower computational load. Devices commonly employed for gesture recognition in IoT emphasize real-time performance and power efficiency. Therefore, the FSK radar is adopted in this paper.

Existing research that adopts radar has only focused on improving the accuracy of hand gesture recognition. Few studies have addressed the decrease in accuracy due to changes in the distance between the user and the radar. This point is important for the real user. Furthermore, most of the research only utilized the CW radar or the FMCW radar for gesture recognition, not the FSK radar. The employment of the FSK radar benefits from the low signal processing computational load of the CW radar and the distance measurement advantages of the FMCW radar. Therefore, this paper presents a novel contribution compared to existing methods.

Furthermore, the proposed algorithm can be applied to various fields. It is especially valuable for drone control in warfare situations, where illumination and power are limited. Because radar has robust characteristics related to the surrounding environment, adopting the FSK radar has benefits in terms of the power consumption.

The remaining sequence of this paper is composed as follows. A micro-Doppler system, Doppler radar sensors, and a micro-Doppler signature signal are introduced in Section 2, and then the CW and FSK radar sensors are compared. Section 3 describes an existing Doppler radar real-time hand gesture recognition system. A hand gesture recognition system using the FSK radar is proposed in Section 4, and Section 5 explains the five methods of the CNN training and inference in the system. Section 6 proposes a preprocessing method to improve the model’s performance. The experimental setting is shown in Section 7, and the results are presented in Section 8. Finally, the paper is summarized and concluded in Section 9.

## 2. Micro-Doppler System

The basic operating principle of a radar sensor is to measure the speed and distance of an object by emitting a microwave signal toward an object and receiving the signal reflected by the object. A moving object changes the frequency of a reflected signal by Doppler effects, and the radar sensor can detect the moving object by the Doppler frequency estimation [19,20,21].

**Figure 1:**
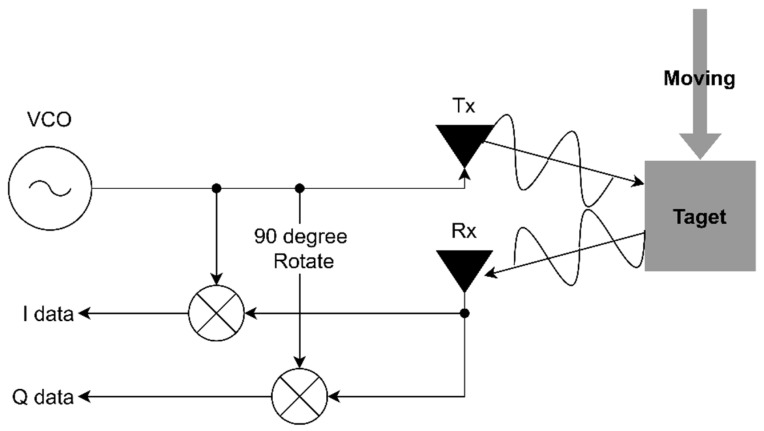
The process of extracting data using the transmit frequency and the receive frequency in a CW radar sensor.

Figure 1 shows the principle of a CW Doppler radar sensor. The Doppler effects occur when either a wave source or a target is in motion. The magnitude of a frequency difference between the emitted signal and the reflected signal is proportional to the relative velocity between the wave sensor and the target [22,23,24]. Figure 1 illustrates that the radar sensor is fixed, so only the target movement can generate the Doppler frequency. A voltage-controlled oscillator (VCO) generates a carrier wave, and the corresponding signal is transmitted via the TX antenna, which goes into a mixer at the same time. Both the transmission and the reflected waves, with frequencies of $f_{T_{X}}$ and $f_{R_{X}}$, are fed into the mixer and mixed into the Doppler frequency.

A micro-Doppler signature refers to the time-varying frequency modulation where a transmitted signal is reflected at a moving point of a target [25,26,27]. The frequency estimated by the radar sensor changes over time in regard to a moving object. The micro-Doppler signature refers to a unique characteristic with respect to a distinguished change in the frequency that is caused by the movement of a target. Micro-Doppler signatures have been researched in various fields, which include human behavior, biosignals, and distinction from animals, and they are widely utilized in real life [28,29].

A micro-Doppler signature can be analyzed from a spectrogram, which is obtained by the short-time Fourier transform (STFT) process. It is generally difficult to find out the overall pattern change in the frequency over time when the frequency of a signal changes with the passage of time. A large part of the temporal change does not appear in the calculated spectrum for the long-time captured nonperiodic signal, so it is advantageous to use the STFT technique, which continuously analyzes a short section where the spectrum component does not change. The STFT repeatedly performs the fast Fourier transform (FFT) process for a moving window. A spectrogram can be formed from the FFT result calculated for each time that can be expressed in three-dimensions (time, frequency, and magnitude).

**Figure 2:**
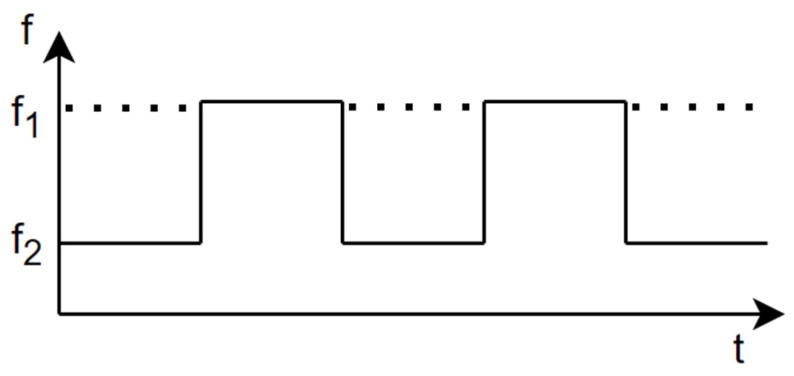
Time–frequency representation of FSK radar.

Figure 2 depicts that the FSK radar transmits the signal by switching two carrier frequencies, $f_{1}$ and $f_{2}$. Switching rapidly occurs and the gap of the two frequencies is very small. The operational principle of each frequency is similar to the CW doppler radar system. However, it can estimate the target distance due to using two frequencies. More detailed explanations will be provided in Section 4 regarding the FSK radar.

## 3. Existing Hand Gesture Recognition Systems

**Figure 3:**
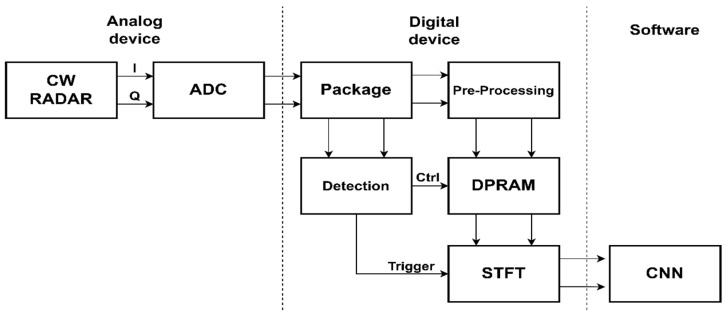
A block diagram of a typical real-time hand gesture recognition system.

Hand gesture recognition means classifying and recognizing specific actions by measuring the data of moving hand gestures, extracting unique features, and analyzing them. The data of hand gestures can be measured using various sensors. A CW radar sensor is conventionally used for gesture recognition. Figure 3 shows a block diagram of a hand gesture recognition system using a CW Doppler radar sensor. The hand gesture recognition system is divided into an analog signal processing section, a digital signal processing section, and a software section. The analog signal processing section measures the micro-Doppler signal of the hand gestures by using a CW radar sensor. A micro-Doppler signal generates both the in-phase (I) and the quadrature-phase (Q) data. The I/Q data are digitalized using an analog-to-digital converter (ADC), which is then fed into the digital signal processing section. The received raw data are adjusted to the required sampling rate via the decimation process in the package block of the digital signal processing section. The preprocessing that is used in order to merge the adjusted I/Q signals is followed for easier data management in the dual-port random access memory (DPRAM). The reorganized data are addressed in a round-robin method and stored in the DPRAM space. The detection module continuously finds a valid frame during this process by analyzing the adjusted signals in real-time. The valid frame means a dataset that consists of useful data samples captured by radar sensors during the motion of the hand gesture. When the detection module claims to have found the valid frame, trigger and control signals are sent to the DPRAM and STFT module. The STFT module reads the valid data stored in the DPRAM and then creates a spectrogram for a software section. The software section analyzes the micro-Doppler signature by using the spectrogram for the hand gesture recognition. The convolutional neural network (CNN) inference is generally performed on hardware accelerators or graphics processing units (GPUs) for the recognition.

It is important to detect a valid frame for a real-time hand gesture system. If the valid data of a hand gesture are not detected properly, the spectrogram that does not contain a meaningful pattern is fed into a neural network, which results in a severe performance degradation with respect to the recognition rate. In an existing method [30], the trigger point is found by the average of the difference between the adjacent time samples in a frame by using Equation (1).

(1) $$T\left[i\right]=\frac{1}{S}\sum_{k=0}^{S-1}\left|x\left[i-k\right]-x\left[i-k-1\right]\right|$$

where S is the frame size, which is set as the number of time samples that cover the motion duration of a hand gesture. Because a frame is moving sample-by-sample, a frame works like a moving window. Here, $\left|x\left[i\right]\right|$ denotes the magnitude of x[i]; T[i] is a trigger value of the i-th frame, which is compared to the predefined threshold value for the frame detection. The i-th frame consists of the i-th time sample and the previous S-1 samples. If the trigger value is smaller than the threshold, the trigger value is recalculated in the next frame. Otherwise, the current frame is declared as a valid frame. This method has as a limit the performance degradation with respect to the detection probability, which is due to both the use without noise estimation and the use of a partial magnitude of a signal, which heavily depends on the motion range of a hand gesture and the noise level.

Another approach [31] determines a valid frame by using the change rate of the trigger value, which consists of the two steps. The first step detects the approximate frame position, like the existing method [30]; the second step verifies the previous decision by using the change rate of the trigger value. The change rate of the trigger value is defined as the trigger ratio, which is calculated using Equation (2).

(2) $$R\left[i\right]=\frac{T\left[i+∆d\right]}{T\left[i\right]}$$

where T[i + ∆d] means the delayed trigger value. The trigger ratio is the ratio of the trigger value of the current frame to the trigger value of the previous frames before a specific time. The trigger ratio can be used in order to find the starting and ending points of the hand gestures. The method using the trigger ratio demonstrates a higher probability of detecting a valid frame and robustness to noise compared to the conventional method utilizing the trigger value. This is attributed to the identification of actual gestures based on the rate of change in the trigger values.

## 4. Proposed Hand Gesture Recognition System Using the FSK Radar

The distance information is hardly obtained in CW radar sensors. In addition, the minimum or maximum detection range of the radar sensors is limited, because it varies depending on both the transmit power and the specifications of the radar sensors. Varying the received power of the radar sensors caused by the change in the motion distance makes accurate hand gesture recognition difficult in the existing hand gesture recognition system utilizing the CW radar sensors. The sensing position of a hand gesture was fixed in previous studies due to this problem. This paper proposes a system adopting the FSK radar sensor that can acquire a comparable gesture-recognition rate, regardless of the motion distance of the hand gestures, by utilizing the distance information.

**Figure 4:**
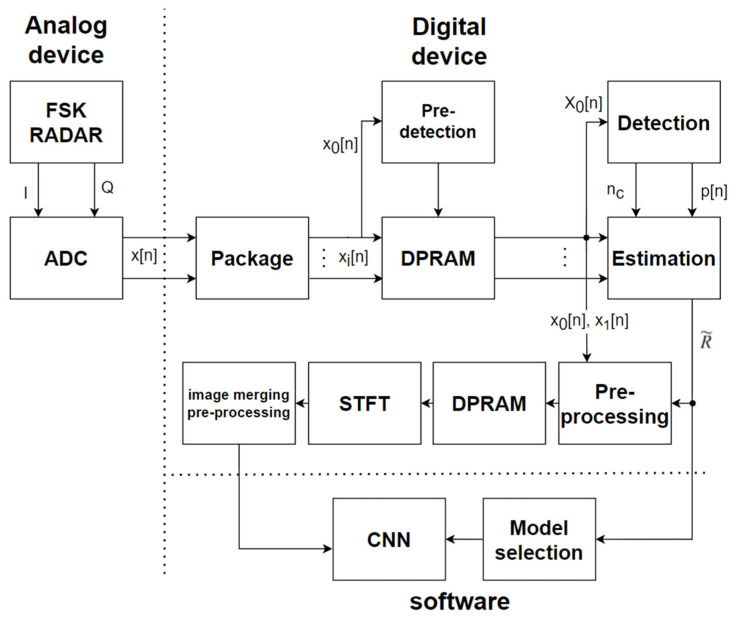
Block diagram of the proposed hand gesture recognition system.

Figure 4 is a block diagram of the proposed real-time hand gesture recognition system that uses the FSK radar sensor. The proposed system is divided into an analog section, a digital section, and a software section. The analog block receives the analog signal that is measured by the FSK radar, and then converts it into I/Q digital time samples for the digital device. The data packaging first adjusts the sampling speed that is required by the system via the decimation process in the digital device. Since the FSK radar sensor alternately transmits two carrier frequencies, the samples are supposed to be divided according to the changing period of the carrier frequency. The received samples that correspond to the same transmit carrier frequency are collected during the data package process based on the frequency-changing period. The system does not know the distance information, and it should save power by preventing wasteful transactions for the motionless state, so a predetection is needed in order to coarsely find a valid frame by using the main data stream, $x_{0}\left[n\right]$. The predetection is similar to the existing algorithms [30]. The data stored in the DPRAM are transferred to the range estimation block whenever the predetection module declares the coarse detection. The detection module searches for the exact valid frame and then sends the center position of the valid frame, which is denoted as $n_{c}$, and the frequency indices, p[n], to the estimation module. The FSK radar sensor adopts two carrier frequencies, which is unlike a CW radar sensor that uses a single carrier frequency, and it utilizes the phase difference of the received signal in order to measure the distance [32]. The distance information estimated in the estimation module, $\tilde{R}$, is utilized for the CNN inference. The stored data streams in the DPRAM are normalized based on the received power in the preprocessing module, which are fed into the other DPRAM. A spectrogram calculation of the valid frame, which is denoted as $X_{0}\left[k,l\right]$, is performed in the STFT module, and the image-merging preprocessing is performed. This is based on the characteristics of the FSK radar, and a further explanation will be provided in Section 6. The output spectrogram is inferred by using a CNN processor with the estimated distance. The CNN processor is separately trained by using the hand gesture datasets, which consist of the classified spectrograms that are generated while changing the distance.

**Figure 5:**
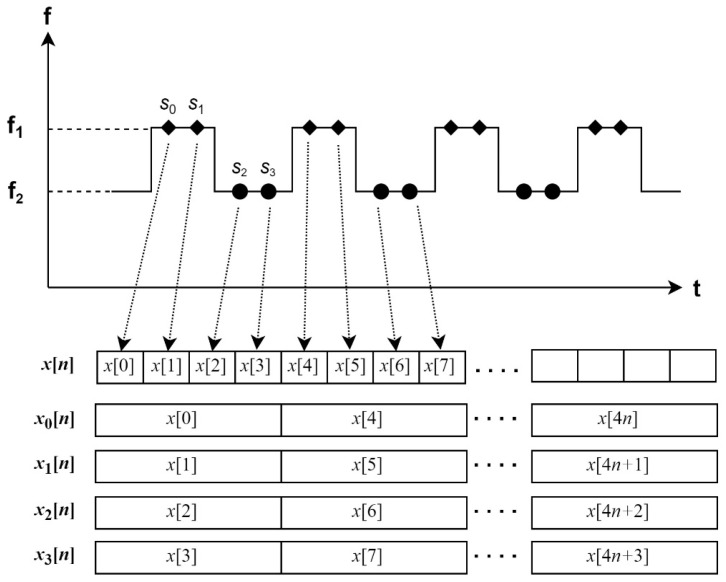
An example of the data sample classification of a received signal in the FSK radar sensor ($S_{S}\;$= 2).

The FSK radar sensor transmits two carrier waves by turns, which have $f_{1}$ and $f_{2}$ frequencies. Figure 5 displays an example of the data sample classification method of a received signal in the FSK radar sensor. When assuming that there are four sample positions of a received signal, $S_{0}$, $S_{1}$, $S_{2}$, and $S_{3}$, during every transmission of two carrier waves, the first data stream, $x_{0}[n]$, is sampled at every $S_{0}$ point, and the second one, $x_{1}[n]$, is sampled at every $S_{1}$ point. The first data stream, $x_{0}[n]$, is mainly used for the frame detection and recognition, and the other streams are used for the image-merging preprocessing. The relation between the output data stream of ADC, which is denoted as x[n], and the classified data streams, $x_{i}[n]$, are defined by Equation (3).

(3) $$x_{i}\left[n\right]=x\left[2\times S_{S}\times n+i\right]$$

where $S_{S}$ is the number of sample positions per each carrier wave. Increasing the number of sample positions per each carrier wave and the samples per each data stream can obtain accurate hand gesture recognition, but it increases the number of FFT points and FFT operations, which require heavy computing power. A decimation process as well as classification are also needed for a reasonable cost of computing power, which can be properly defined by the performance and objectives of a sensor system.

**Figure 6:**
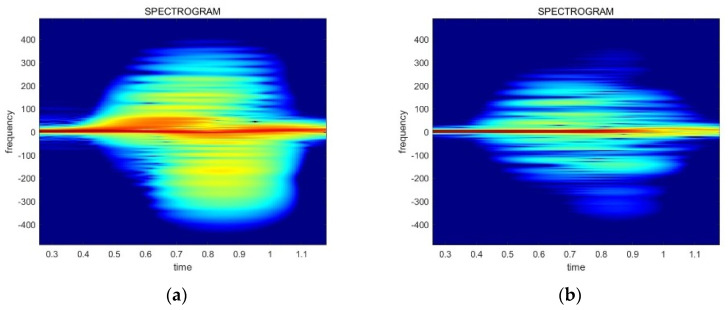
The spectrogram images generated from data collected at various power levels: (**a**) a spectrogram of a high received power signal; (**b**) a spectrogram of a low received power signal.

There are differences among the spectrograms based on the received power. Figure 6 illustrates the difference between the two types of data, which have an influence on the CNN model training and inference. Thus, the power normalization preprocessing has to be conducted for effective neural network training and recognition accuracy. The normalization equations are provided in Equations (4) and (5).

(4) D_N[n]=G×D[n]

(5) G=P_const/P_avg

**Figure 7:**
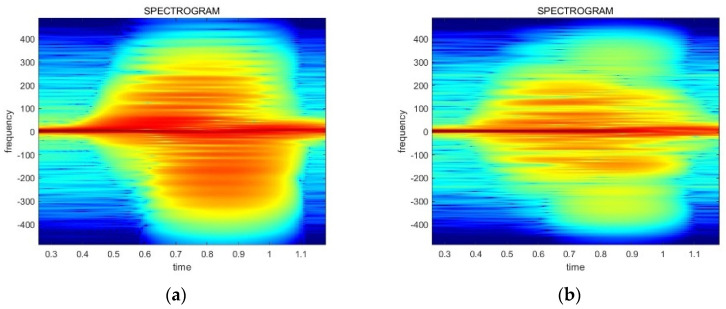
The result of the power normalization related to the data in Figure 6: (**a**) a normalized spectrogram of a high received power signal; (**b**) a normalized spectrogram of a low received power signal.

D[n] represents the original radar data and D_N[n] is the normalized data. G denotes the gain value, P_avg is the average power value of the original data, and P_const refers to the power normalization coefficient, which is set to $10^{6}$ in this paper. The result of the power normalization is displayed in Figure 7. Finally, these preprocessed spectrograms are used for training and inferencing the gestures.

## 5. Proposed CNN Training and Inference Methods

This paper introduces five methods for creating a distance-adaptive hand gesture recognition system using the FSK radar. One is the proposed method in this paper, while the remaining four methods are the candidates that we explored. The system aims to cover a range from 30 cm to 180 cm. To train the CNN models for these methods, we collected training data at 30 cm intervals—specifically, data measured at distances of 30, 60, 90, 120, 150, and 180 cm. Also, the model was tested using data measured at 10 cm intervals.

**Figure 8:**
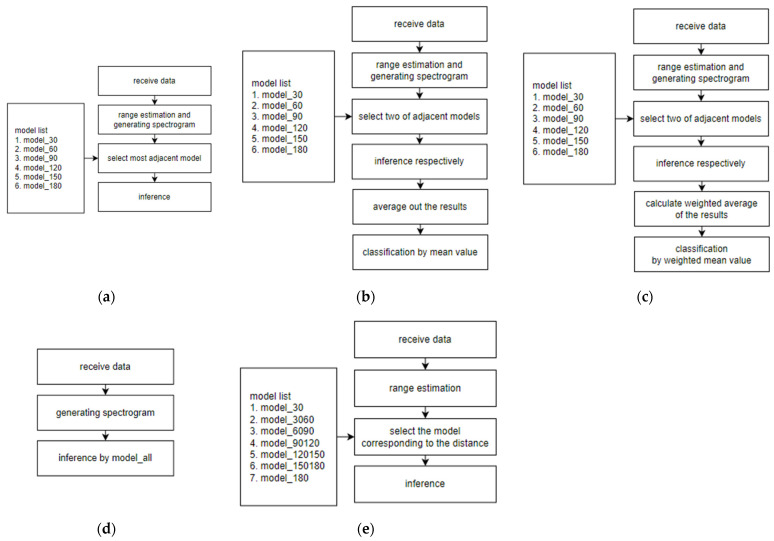
Block diagram of the introduced methods: (**a**) candidate 1; (**b**) candidate 2; (**c**) candidate 3; (**d**) candidate 4; (**e**) proposed method.

The proposed method and candidates 1, 2, and 3 train multiple models via the training data, and the proper model was selected by utilizing the estimated distance from the FSK radar. On the other hand, candidate 4 combines the data from various distances and trains with the combined dataset. Figure 8 displays block diagrams of each method, and the detail of each method will be explained.

The common CNN model structure used in the explored methods is summarized in Table 1. The neural network in this paper has advantages in terms of complexity. Gesture recognition systems, commonly employed in environments such as smart homes and edge devices, require a low power consumption and computational speed for real-time operation. This paper considered the number of parameters so to choose its own CNN model instead of traditionally renowned models, which include ResNet, MobileNet, and EfficientNet. The increase in the parameter count induces a rise in computational resources and a degradation in the inference speed. Furthermore, models with many parameters are unsuitable for edge devices because they demand significant memory. Additionally, the memory read and write operations are time-consuming tasks with considerable power consumption. Therefore, a model with fewer parameters is advantageous for a hand gesture recognition system. Table 2 presents a comparison of the total parameters between our CNN model and classical models. We compared our CNN model with ResNet18, which has the fewest layers among the ResNet variants, and with EfficientNetB0, the lightest version of the EfficientNet architecture.

### 5.1. Candidate 1

Candidate 1 involves training models corresponding to each distance by utilizing data that are measured at each distance. A total of six trained models are obtained with the training data that are available at 30 cm intervals. The key point is selecting the appropriate model based on the estimated distance information from the radar. In this method, the nearest model from the estimated distance is chosen. For instance, if the distance is 40 cm, the 30 cm model, which is the closest from the estimated distance, will be selected.

### 5.2. Candidate 2

Candidate 2 also trains six models with the corresponding distance. However, the difference lies in the inference stage. This method uses two models that are adjacent to the estimated distance. First, the method conducts inference with the two models, respectively. For example, if the estimated distance is 50 cm, the 30 cm model and 60 cm model will be chosen for the inference. The predicted values from each model are obtained, averaged, and the final recognition decision is based on the highest average value across all classes.

### 5.3. Candidate 3

Candidate 3 also involves training models for each distance, which is similar to the previous methods. As in candidate 2, adjacent models are used during the inference. However, the key difference lies in the handling of the results of the inference. Candidate 3 utilizes a weighted average based on the distance instead of averaging the probability of the adjacent models. For instance, if the measured distance is 40 cm, a higher weight is assigned to the 30 cm model, whereas a lower weight is assigned to the 60 cm model. After that, the weighted average is conducted. Equation (6) expresses the weighted average.

(6) $$weighted\;mean\;value=pred\_1\times \frac{\left(30-R\right)}{30}+pred\_2\times \frac{R}{30}$$

Here, pred_1 represents the prediction of the model closer to the radar between the two adjacent models, while pred_2 is the prediction farther away from the model.

### 5.4. Candidate 4

Candidate 4 integrates the entire training dataset into a single set. In this case, inference is conducted regardless of the estimated distance.

### 5.5. Proposed Method

The proposed method involves training models by combining the training data from adjacent distances. Specifically, data from 30 cm and 60 cm are merged into one training set, followed by merging data from 60 cm and 90 cm into another training set. Plus, datasets that consist of only 30 cm and 180 cm are utilized in order to cover the distance from 0 to 30 cm and beyond 180 cm. A total of seven trained models are obtained. The distance information is used to select the appropriate model during the inference. For instance, if the distance is measured as 40 cm, the model trained on the combined dataset of 30 cm and 60 cm is chosen. Also, if the estimated distance is 110 cm, the model that was trained on the combined dataset of 90 cm and 120 cm is selected.

## 6. Image-Merging Preprocessing

The data preprocessing method related to the FSK radar system is also proposed in this paper. It is used for the AI training and inference.

**Figure 9:**
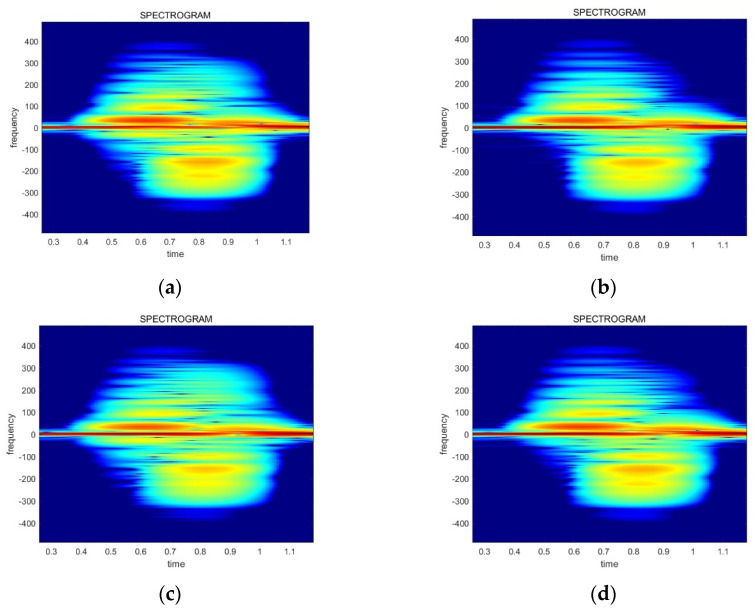
Examples of the phenomenon that shows distinct spectrograms at the same carrier frequency: (**a**) data stream $x_{0}\left[n\right]$ at frequency $f_{1}$; (**b**) data stream $x_{1}\left[n\right]$ at frequency $f_{1}$; (**c**) data stream $x_{2}\left[n\right]$ at frequency $f_{2}$; (**d**) data stream $x_{3}\left[n\right]$ at frequency $f_{2}$.

Figure 5 in Section 4 explains the four data streams in the FSK radar system. The $x_{0}\left[n\right]$ and $x_{1}\left[n\right]$ data streams correspond to the $f_{1}$ frequency, whereas the $x_{2}[n]$ and $x_{3}\left[n\right]$ data streams correspond to the $f_{2}$ frequency. It is expected that the spectrograms of the data streams at the same frequency exhibit the same shape. Consequently, four spectrograms have the same appearance due to the small frequency gap between the two carrier frequencies. However, in real radar data, the spectrograms show different shapes at the same carrier frequency. In other words, $x_{0}\left[n\right]$ and $x_{1}\left[n\right]$ have distinct spectrograms. Similarly, the $x_{2}[n]$ and $x_{3}\left[n\right]$ data streams also show differences despite sharing the same frequency. Figure 9 explains this phenomenon, which has distinct features on the top-right side and the bottom-left side. The original images are used in Figure 9 for the visibility, while the power-normalized images are utilized in the real model training and inference.

A phase-locked loop (PLL) cannot ideally switch instantaneously between two frequencies. It takes time to go into a stable state. If the data stream was captured at an unstable frequency, this phenomenon can occur. In other words, even-numbered data streams are in a stable state and odd-numbered streams are in an unstable state; or, it can be the opposite.

Although commercial radar aims to set the position of the data samples at a stable state, this phenomenon can occur due to slight instabilities. The levels of instability from the PLL vary for each radar, and creating an ideal PLL is challenging. Thus, it is feasible to use this phenomenon in other FSK radar systems by utilizing the data from the corresponding radar.

**Figure 10:**
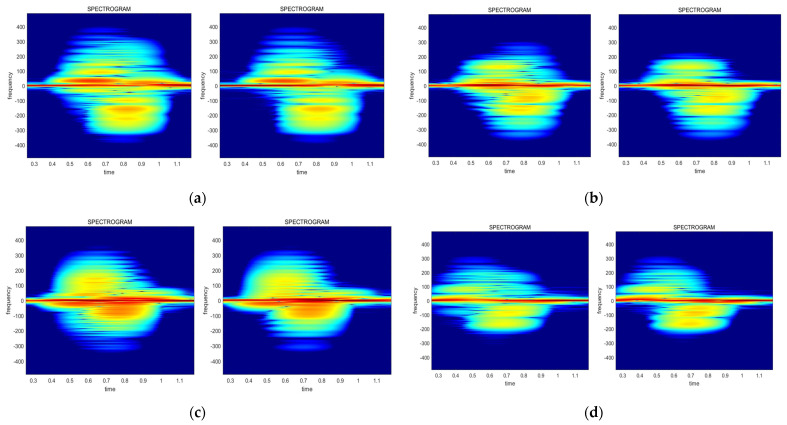
The changing pattern of the spectrograms for each gesture: (**a**) UPtoDOWN; (**b**) RIGHTtoLEFT; (**c**) LEFTtoRIGHT; (**d**) DOWNtoUP.

Similar patterns of each gesture are observed during the spectrogram analysis. Figure 10 illustrates that the UPtoDOWN and LEFTtoRIGHT gestures show relatively small spectrogram differences between each of the data streams, whereas the RIGHTtoLEFT and DOWNtoUP gestures show more distinct appearances. Also, the UPtoDOWN, RIGHTtoLEFT, and DOWNtoUP gestures have more differences at the upper-right side, while the LEFTtoRIGHT gesture displays more changes at the bottom-left side. Consequently, this paper proposes utilizing these patterns for the AI model training in order to improve the recognition accuracy.

**Figure 11:**
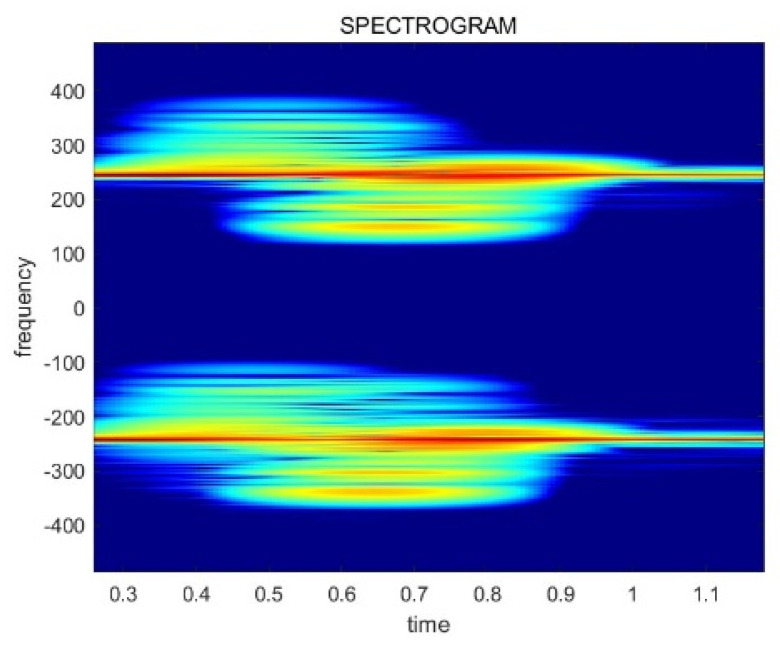
An example of the image merging-data preprocessing.

The CNN shows a high performance in regard to finding the features and patterns of the images. Thus, it is helpful to forcibly generate patterns to improve the CNN performance by merging the spectrogram images from each data stream. This merging-data preprocessing is illustrated in Figure 11.

We conducted experiments by applying this preprocessing method to the proposed FSK radar systems, which are described in Section 5, and compared the results. To ensure a fair comparison, the model structures were kept the same. Therefore, the size of the resulting image was maintained to be the same as that of the original image, even when the images were merged.

## 7. Experiment

**Figure 12:**
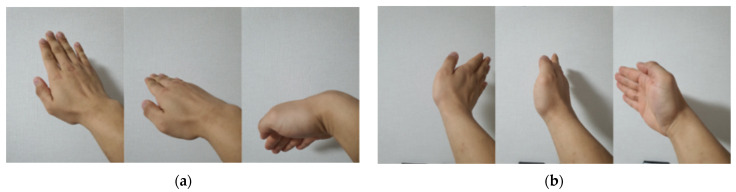

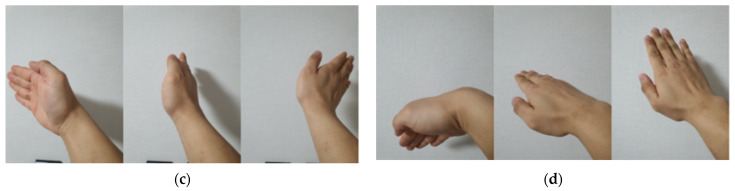
Examples of each gesture. (**a**) UPtoDOWN; (**b**) RIGHTtoLEFT; (**c**) LEFTtoRIGHT; (**d**) DOWNtoUP.

**Figure 13:**
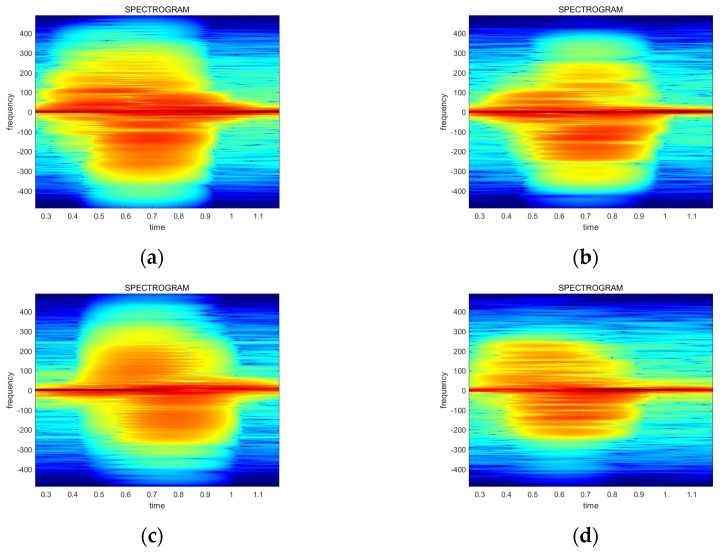
Examples of the spectrograms obtained when the motion for each label was taken. (**a**) UPtoDOWN; (**b**) RIGHTtoLEFT; (**c**) LEFTtoRIGHT; (**d**) DOWNtoUP.

A commercial FSK radar was utilized to verify the performance of the proposed algorithm in this paper, and the system parameters were set up as shown in Table 3. The ADC sampling rate was 125 ksps and the sample time duration of the data stream was 32 us, because the data are classified into four groups by the package block. Figure 12 illustrates the four hand gesture types, including UPtoDOWN, DOWNtoUP, RIGHTtoLEFT, and LEFTtoRIGHT. Figure 13 shows examples of the obtained spectrograms corresponding to each gesture. Eight volunteers participated in the radar measurement. The measurements were taken from participants with varying genders and body sizes to ensure general performance across different users. A total of 230 samples were taken at distances of 30, 60, 90, 120, 150, and 180 cm for each gesture, which were used for the neural network training and testing. Additionally, data at distances of 40, 50, 70, 80, 100, 110, 130, 140, 160, and 170 cm, which were exclusively used for the inference, were captured at 60 samples per gesture. Consequently, a total of 4080 samples were used in the training and 3840 samples were used in the testing. The ratio for the test dataset was higher than usual, given that the inference was conducted for sixteen distances.

Four hand gestures were tested with the proposed methods by using the FSK radar sensor. Additionally, the test included the case not using the distance information in the CW radar system. The time indices of the ideal time positions were manually checked for every dataset for the test of the valid frame detection. The other system parameters were experimentally selected to achieve the best system performance.

## 8. Result

### 8.1. Result of the Existing Method

**Figure 14:**
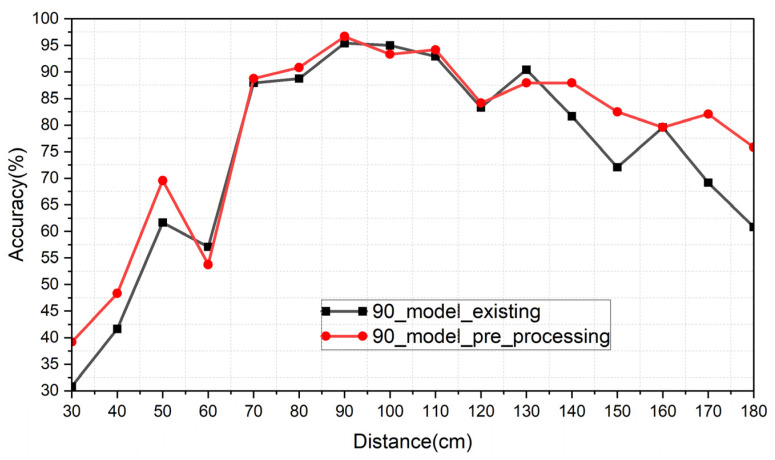
Graph of Table 4.

Table 4 summarizes the inference result of the existing CW radar system. The 90 cm model, which was positioned at the center of 30~180 cm, was used to assess the recognition accuracy across the entire range of distances. The table also includes the results of the image-merging preprocessing that was applied to the 90 cm model. In this case, the FSK radar was utilized due to the preprocessing. Figure 14 shows the graph of Table 4. The inference at 90 cm showed the highest accuracy, which decreased as the distance increased from 90 cm. Furthermore, it exhibited an average accuracy that was 4.14% higher compared to the case without the preprocessing when applying the image-merging preprocessing.

As a result, users have to keep their fixed position to achieve a better recognition performance in the existing method. Therefore, this system cannot cover a wide range. On the other hand, the preprocessing demonstrates that it improves the recognition accuracy.

### 8.2. Result of the Proposed Method

The experiments for the proposed method and candidates were conducted for four different scenarios. The experiments were divided into two cases, which include one with the image-merging preprocessing and another without it. Each of these two cases was further divided into two subcases based on whether the real distance information, which was estimated from the FSK radar, or an ideal distance was used. These four scenarios are summarized in Table 5.

**Figure 15:**
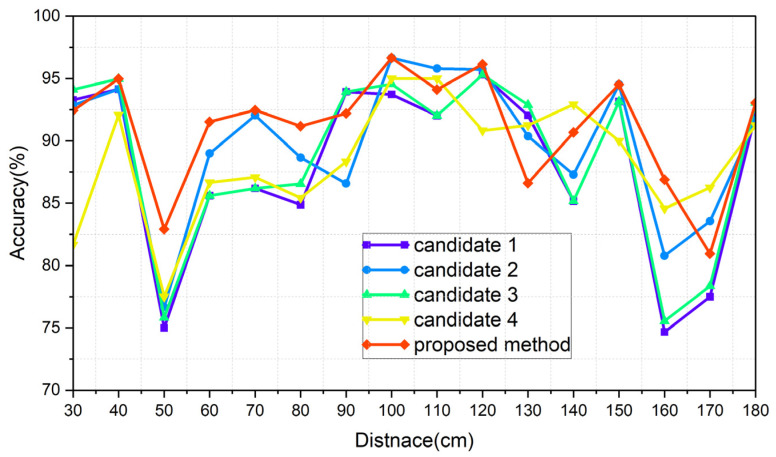
Graph of scenario Normal_real.

**Figure 16:**
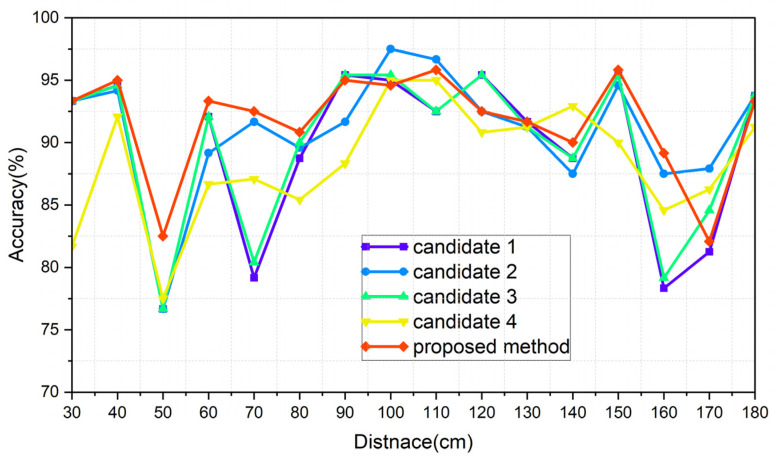
Graph of scenario Normal_ideal.

**Figure 17:**
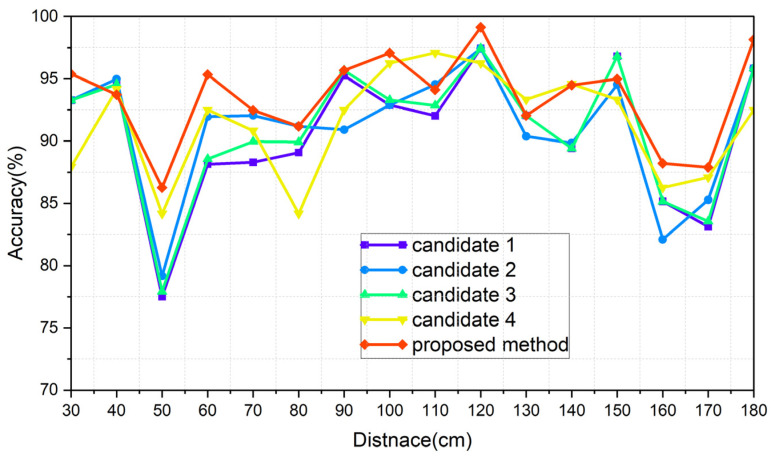
Graph of scenario Merging_real.

**Figure 18:**
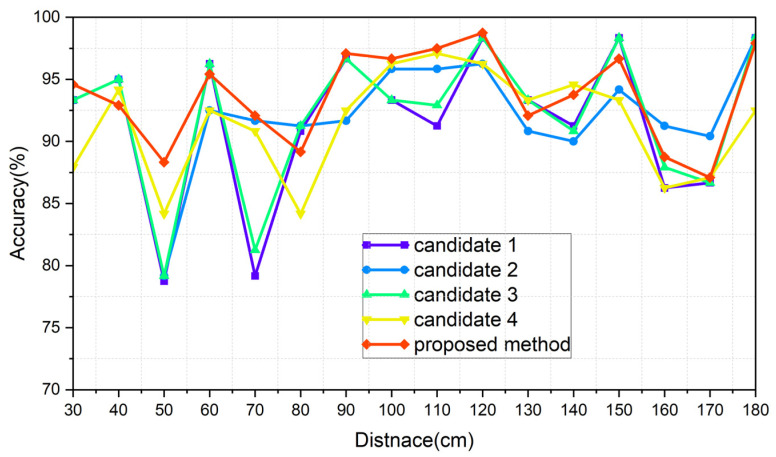
Graph of scenario Merging_ideal.

The results of each of the scenarios are indicated in Table 6, Table 7, Table 8 and Table 9, respectively, and Figure 15, Figure 16, Figure 17 and Figure 18 illustrate the graphs that correspond to Table 6, Table 7, Table 8 and Table 9. The analysis of each result is conducted in Section 8.3.

### 8.3. Comparative Analysis of the Results of the Introduced Methods

The average accuracy for each distance was used as a performance metric. Table 10 shows the comparison of the Normal_real and Normal_ideal cases. The methods include the distance information, except for candidate 4, which improved the accuracy when the ideal distance was utilized. The performance improvement of the proposed method was relatively low among them. The reason for this is that the proposed method exhibits an intermediate characteristic between candidate 4 and candidates 1, 2, and 3. A smaller gap between the two cases results in a more reliable system because it means that the system is less influenced by the distance-estimation algorithm. This robustness also implies an advantage in the scalability across various fields, as there is less need to consider the distance estimation. Therefore, the proposed method and candidate 4 offer benefits in creating a more robust system.

A comparison between Merging_real and Merging_ideal is summarized in Table 11. In the case of applying preprocessing, the accuracy gap, similar to the normal case, is also small for the proposed method and candidate 4 with respect to the distance information.

Table 12 and Table 13 show a comparison of the differences between applying and not applying the image-merging preprocessing for both the real distance and ideal distance cases, respectively. It was demonstrated that the preprocessing improves the recognition accuracy for every case. Therefore, it is appropriate to apply the mentioned preprocessing when using the FSK radar.

Figure 15, Figure 16, Figure 17 and Figure 18 show the worst accuracy at the distance of 50 cm. This paper was given training data from distances of 30, 60, 90, 120, 150, and 180 cm. It is normal that the performance of the intermediate distances might not be high, because the intermediate distances are supplemented with an algorithm using the given data. For distances of 90 cm and 120 cm, as well as 120 cm and 150 cm, the patterns between the training datasets show less variation compared to other areas, resulting in a high similarity and less degradation in the inference performance in ungiven areas. During the experiments, even when the same participant performed the same action repeatedly, pattern differences could occur. Distances like 30 and 60 cm, 60 and 90 cm, and 150 and 180 cm exhibited significant pattern differences between training datasets, while 90 and 120 cm and 120 and 150 cm showed less pattern difference. This phenomenon is observed in Figure 15, Figure 16, Figure 17 and Figure 18. To properly validate the effectiveness of the proposed method in the depicted figures, it is crucial to examine the regions with substantial pattern differences between training datasets (i.e., the intervals between 30 and 60 cm, 60 and 90 cm, and 150 and 180 cm). The proposed method demonstrates a significant mitigation for performance degradation compared to other candidates in these areas, indicating its effectiveness as a highly efficient approach. Consequently, the proposed method showed the highest performance in every scenario, which is summarized in Table 14.

The conventional method using the CW radar demonstrated a maximum hand gesture recognition accuracy of 94.21% [31]. However, when the proposed method and preprocessing techniques were applied, it achieved an accuracy of 93.67%, maintaining a similar level of accuracy while covering a wide range of distances.

## 9. Conclusions

The FSK radar sensor system for real-time hand gesture recognition was proposed in this paper. The proposed system utilized the dataset-adjustment scheme depending on the distance information. The existing methods adopting CW radar sensors could not deal with the variance of the received signal caused by the change in the distance, whereas the proposed method could maintain a reasonable recognition performance due to the dataset correction depending on the change in the distance. This made sense, because the CNN model could be trained and tested by using the useful dataset with distinguishable patterns regardless of the distance. This paper also proposed a spectrogram image preprocessing method using the characteristics of the FSK radar in order to enhance the gesture-recognition accuracy. The deep-learning performance was improved due to the new features that distinguished each gesture by adopting the image preprocessing.

A commercial FSK radar sensor was utilized for the experiments. The hand gestures consisted of a total of four movements, and the distances between the user and the radar sensor were from 30 cm to 180 cm with spaces every 10 cm. The labels were defined for each movement and distance. The recognition and classification probabilities were defined based on the CNN model, which was trained based on the data.

It was found from the experimental results that (1) adopting the existing system for covering the wide range is unsuitable. The average accuracy was 74.27%, and it showed at most 78.41%, even when utilizing the proposed preprocessing method. (2) The recognition probability of the proposed method was the highest in every scenario. It was 93.51% with the real estimated distance, and 93.67% with the ideal value. (3) The proposed image-merging preprocessing increased the accuracy in all cases.

The proposed real-time hand gesture recognition system showed considerable recognition performance even under the change in distance, which is useful for various application systems that require effective and secure human–computer interaction techniques. In future work, we plan to improve the degradation in the recognition accuracy when the hand gestures deviate in the vertical or horizontal directions, rather than occurring directly in front of the radar. As another research direction, we are contemplating studies aimed at dealing with variations in the recognition results from the differences in the execution speed of the human hand gestures.

## Figures and Tables

**Table 1:** Specifications of the CNN model structure used for hand gesture recognition.

Layer	Output Shape
input	656 × 875 × 3
Conv1 + batch normalization+ ReLU + max pooling	328 × 437 × 16
Conv2 + batch normalization + ReLU + max pooling	164 × 218 × 32
Conv3 + batch normalization + ReLU + max pooling	82 × 109 × 64
Conv4 + batch normalization + ReLU + max pooling	41 × 54 × 128
Conv5 + batch normalization + ReLU	41 × 54 × 256
Fully connected layer	4

**Table 2:** A comparison of the number of the total parameters between the paper’s own CNN model and classical models.

Model	CNN in This Paper	ResNet18	MobileNet	EfficientNet
Total parameters	947,556	21,069,956	4,282,564	5,365,415

**Table 3:** Experimental specifications.

RADAR Specifications and System Parameters	Descriptions
Radar model	K-MC1 [33]
ADC sampling rate	125 ksps
$S_{S}$	2
TS	32 us (=1/31.25 ksps)
∆f	9 MHz
$f_{1}$	24.125 GHz
$f_{2}$	24.134 GHz
Hand gesture types	UPtoDOWN, RIGHTtoLEFT, LEFTtoRIGHT, DOWNtoUP
Distance from the sensor	10 cm interval from 30 cm to 180 cm
Number of datasets per each label for the CNN	1980
Number of test participants	8

**Table 4:** The recognition accuracy of the existing method and the case of adopting the proposed data preprocessing on an existing method.

Distance (cm)	Model_90 (Existing Method)	Model_90 with Merging Preprocessing
30	30.83%	39.17%
40	41.67%	48.33%
50	61.67%	69.58%
60	57.08%	53.75%
70	87.92%	88.75%
80	88.75%	90.83%
90	95.42%	96.67%
100	95.00%	93.33%
110	92.92%	94.17%
120	83.33%	84.17%
130	90.42%	87.92%
140	81.67%	87.92%
150	72.08%	82.50%
160	79.58%	79.58%
170	69.17%	82.08%
180	60.83%	75.83%
average	74.27%	78.41%

**Table 5:** The four scenarios for the experiments of the introduced method.

Scenario Name	Description	
	Image-Merging Preprocessing	Distance Information
Normal_real	No	Real value
Normal_ideal	No	Ideal value
Merging_real	Yes	Real value
Merging_ideal	Yes	Ideal value

**Table 6:** The results of scenario Normal_real.

Distance (cm)	Candidate 1	Candidate 2	Candidate 3	Candidate 4	Proposed
30	93.28%	92.86%	94.12%	81.67%	92.44%
40	94.14%	94.14%	94.98%	92.08%	94.98%
50	75.00%	76.67%	75.83%	77.50%	82.92%
60	85.59%	88.98%	85.59%	86.67%	91.53%
70	86.19%	92.05%	86.19%	87.08%	92.47%
80	84.87%	88.66%	86.55%	85.42%	91.18%
90	93.94%	86.58%	93.94%	88.33%	92.21%
100	93.72%	96.65%	94.56%	95.00%	96.65%
110	92.02%	95.80%	92.02%	95.00%	94.12%
120	95.30%	95.73%	95.30%	90.83%	96.15%
130	92.05%	90.38%	92.89%	91.25%	86.61%
140	85.17%	87.29%	85.17%	92.92%	90.68%
150	93.15%	94.52%	93.15%	90.00%	94.52%
160	74.67%	80.79%	75.55%	84.58%	86.90%
170	77.49%	83.55%	78.35%	86.25%	80.95%
180	92.13%	91.67%	93.06%	91.25%	93.06%
average	88.05%	89.77%	88.58%	88.49%	91.08%

**Table 7:** The results of scenario Normal_ideal.

Distance (cm)	Candidate 1	Candidate 2	Candidate 3	Candidate 4	Proposed
30	93.33%	93.33%	93.33%	81.67%	93.33%
40	94.58%	94.17%	94.58%	92.08%	95.00%
50	76.67%	76.67%	76.67%	77.50%	82.50%
60	92.08%	89.17%	92.08%	86.67%	93.33%
70	79.17%	91.67%	80.42%	87.08%	92.50%
80	88.75%	89.58%	90.00%	85.42%	90.83%
90	95.42%	91.67%	95.42%	88.33%	95.00%
100	95.00%	97.50%	95.42%	95.00%	94.58%
110	92.50%	96.67%	92.50%	95.00%	95.83%
120	95.42%	92.50%	95.42%	90.83%	92.50%
130	91.67%	91.25%	91.25%	91.25%	91.67%
140	88.75%	87.50%	88.75%	92.92%	90.00%
150	95.42%	94.58%	95.42%	90.00%	95.83%
160	78.33%	87.50%	79.17%	84.58%	89.17%
170	81.25%	87.92%	84.58%	86.25%	82.08%
180	93.75%	93.75%	93.75%	91.25%	93.33%
average	89.51%	90.96%	89.92%	88.49%	91.72%

**Table 8:** The results of scenario Merging_real.

Distance (cm)	Candidate 1	Candidate 2	Candidate 3	Candidate 4	Proposed
30	93.28%	93.28%	93.28%	87.92%	95.38%
40	94.56%	94.98%	94.56%	94.17%	93.72%
50	77.50%	79.17%	77.92%	84.17%	86.25%
60	88.14%	91.95%	88.56%	92.50%	95.34%
70	88.28%	92.05%	89.96%	90.83%	92.47%
80	89.08%	91.18%	89.92%	84.17%	91.18%
90	95.24%	90.91%	95.67%	92.50%	95.67%
100	92.89%	92.89%	93.31%	96.25%	97.07%
110	92.02%	94.54%	92.86%	97.08%	94.12%
120	97.44%	97.44%	97.44%	96.25%	99.15%
130	92.05%	90.38%	92.05%	93.33%	92.05%
140	89.41%	89.83%	89.41%	94.58%	94.49%
150	96.80%	94.52%	96.80%	93.33%	94.98%
160	85.15%	82.10%	85.15%	86.25%	88.21%
170	83.12%	85.28%	83.55%	87.08%	87.88%
180	95.83%	95.83%	95.83%	92.50%	98.15%
average	90.67%	91.02%	91.02%	91.43%	93.51%

**Table 9:** The results of scenario Merging_ideal.

Distance (cm)	Candidate 1	Candidate 2	Candidate 3	Candidate 4	Proposed
30	93.33%	93.33%	93.33%	87.92%	94.58%
40	95.00%	95.00%	95.00%	94.17%	92.92%
50	78.75%	79.17%	79.17%	84.17%	88.33%
60	96.25%	92.50%	96.25%	92.50%	95.42%
70	79.17%	91.67%	81.25%	90.83%	92.08%
80	90.83%	91.25%	91.25%	84.17%	89.17%
90	96.67%	91.67%	96.67%	92.50%	97.08%
100	93.33%	95.83%	93.33%	96.25%	96.67%
110	91.25%	95.83%	92.92%	97.08%	97.50%
120	98.33%	96.25%	98.33%	96.25%	98.75%
130	93.33%	90.83%	93.33%	93.33%	92.08%
140	91.25%	90.00%	90.83%	94.58%	93.75%
150	98.33%	94.17%	98.33%	93.33%	96.67%
160	86.25%	91.25%	87.92%	86.25%	88.75%
170	86.67%	90.42%	86.67%	87.08%	87.08%
180	98.33%	98.33%	98.33%	92.50%	97.92%
average	91.69%	92.34%	92.06%	91.43%	93.67%

**Table 10:** A comparison between Normal_real and Normal_ideal.

Scenario	Prop1	Prop2	Prop3	Prop4	Prop5
Normal_real	88.05%	89.77%	88.58%	88.49%	91.08%
Normal_ideal	89.51%	90.96%	89.92%	88.49%	91.72%
gap	1.46%	1.19%	1.34%	0.00%	0.63%

**Table 11:** A comparison between Merging_real and Merging_ideal.

Scenario	Prop1	Prop2	Prop3	Prop4	Prop5
Merging_real	90.67%	91.02%	91.02%	91.43%	93.51%
Merging_ideal	91.69%	92.34%	92.06%	91.43%	93.67%
gap	1.02%	1.32%	1.04%	0.00%	0.17%

**Table 12:** A comparison between Normal_real and Merging_real.

Scenario	Prop1	Prop2	Prop3	Prop4	Prop5
Normal_real	88.05%	89.77%	88.58%	88.49%	91.08%
Merging_real	90.67%	91.02%	91.02%	91.43%	93.51%
gap	2.63%	1.25%	2.44%	2.94%	2.42%

**Table 13:** A comparison between Normal_ideal and Merging_ideal.

Scenario	Prop1	Prop2	Prop3	Prop4	Prop5
Normal_ideal	89.51%	90.96%	89.92%	88.49%	91.72%
Merging_ideal	91.69%	92.34%	92.06%	91.43%	93.67%
gap	2.19%	1.38%	2.14%	2.94%	1.95%

**Table 14:** Accuracy ranking for each of the cases among the introduced methods.

Scenario	1st	2nd	3rd	4th	5th
Normal_real	proposed	candidate 2	candidate 3	candidate 4	candidate 1
Normal_ideal	proposed	candidate 2	candidate 3	candidate 1	candidate 4
Merging_real	proposed	candidate 4	candidate 2	candidate 3	candidate 1
Merging_ideal	proposed	candidate 2	candidate 3	candidate 1	candidate 4

## Data Availability

Data are contained within the article.
